# Anchoretic Infection

**DOI:** 10.1155/2012/263291

**Published:** 2012-11-04

**Authors:** S. Gokkulakrishnan, Ashish Sharma, Satish Kumaran, P. L. Vasundhar

**Affiliations:** ^1^Department of OMFS, Institute of Dental Science Bareilly, Bareilly 243006, India; ^2^Department of OMFS, Kothiwal Dental College & Research Centre, Moradabad, India; ^3^Department of OMFS, Sri Sai College of Dental Surgery, Srikakulam, India

## Abstract

Active and passive mouth opening exercises are a very common practice in oral and maxillofacial surgery especially for various conditions causing limited mouth opening like space infections, trauma, and ankylosis. But most of the practitioners do not follow basic principles while advocating these active mouth opening exercises and also take it for granted that it would benefit the patient in the long run. Because of this, the mouth opening physiotherapy by itself can at times lead to unwanted complications. We report a case wherein due to active physiotherapy, the patient had complications leading to persistent temporal space infection which required surgical intervention and hospitalization. This could have been because of hematoma formation during physiotherapy which got infected due to anchoretic infection of unknown etiology and resulted in temporal space infection. Hence, our conclusion is that whenever mouth opening exercises are initiated, it should be done gradually under good antibiotic coverage to avoid any untoward complications and for optimum results. According to the current English literature, such a complication has not been documented before.

## 1. Introduction

 The term “Trismus” is derived from a Greek word “Trismos” meaning squeaking, whistling, or whizzing [1]. The principal manifestation of trismus is restricted jaw movement that can severely affect nutrition, oral hygiene, and speech and in some cases can result in airway compromise. It is one of the most common complications which can occur due to infection, trauma, temporomandibular joint disorders, MPDS, submucous fibrosis, and so forth. This condition is usually managed conservatively with analgesics, muscle relaxants, hot fomentations, and mouth opening physiotherapy. But sometimes surgical intervention is required which will again be followed by aggressive mouth opening physiotherapy [2].

 In children fibrous ankylosis especially following injury to temporomandibular joint can also manifest as trismus. The most common treatment employed by majority of practioners for this is vigorous mouth opening physiotherapy [3]. This form of treatment is slow but eventually successful, and one can easily achieve good mouth opening. We present an unusual case report of a patient who had temporal space infection as a result of vigorous mouth opening physiotherapy for trismus due to fibrous ankylosis. According to our present knowledge such a case has not been documented anywhere in the literature. 

## 2. Case Report

**Figure 1:**
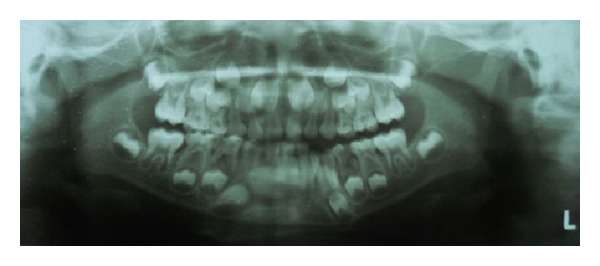
Preoperative OPG.

A seven-year-old male patient from lower socioeconomic strata reported to the Department of Oral and Maxillofacial Surgery with a chief complaint of restricted mouth opening from two and a half months before. The patient gave a history of fall and trauma to the right side of face while playing three months before. Following this, the patient noticed gradual decrease in mouth opening with development of trismus which was not associated with any swelling or pain. On examination, reduced interincisal distance (12 mm) with restricted condylar movement of the right side was noticed. Panoramic radiograph (OPG) was advised, but nothing significant was revealed from it apart from carious 74 (Figure 1). A diagnosis of fibrous ankylosis of the right temporomandibular joint was reached. 

 Considering the patient's age, diagnosis of fibrous ankylosis, application of force full mouth opening under general anesthesia followed by both active and passive vigorous physiotherapy was planned. Mouth opening of 32 mm was achieved after forceful mouth opening under general anesthesia. The patient was discharged on the next day, and the patient's parents were advised to continue mouth opening physiotherapy using Heister's mouth gag.

**Figure 2:**
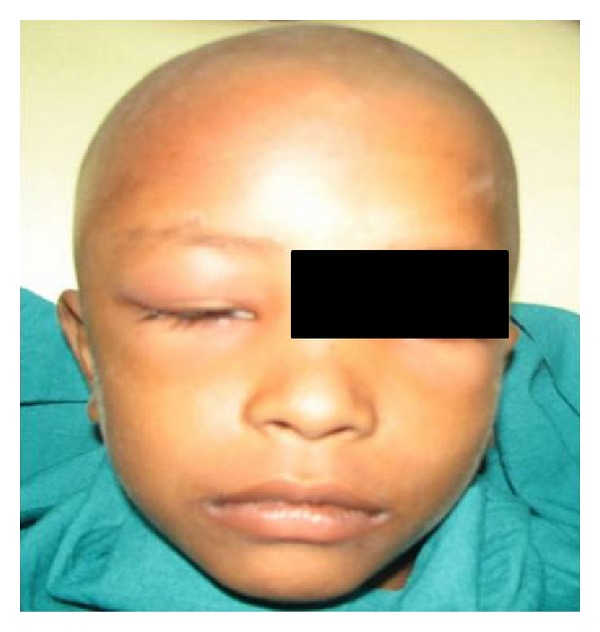
Swelling over the temporoorbital region.

 2 weeks later the patient reported back with complaint of restricted mouth opening associated with pain and swelling over the right temporoorbital region (Figure 2). Swelling was fluctuant, tender with rise in local temperature, and indicating towards infection of temporal region. No constitutional symptoms were noted and no correlation to any odontogenic cause for infection was found. The patient was further referred to an ophthalmologist, a neurologist, and an ENT surgeon for ruling out any eye, ear, nose, tonsil, cranial, or sinus infections which could be related to the infection over the temporal region. 

The patient was treated with incision and drainage via extraoral approach under local anesthesia with empirical antibiotic coverage. Pus was sent for culture and sensitivity. Mild mouth opening physiotherapy was again initiated.

**Figure 3:**
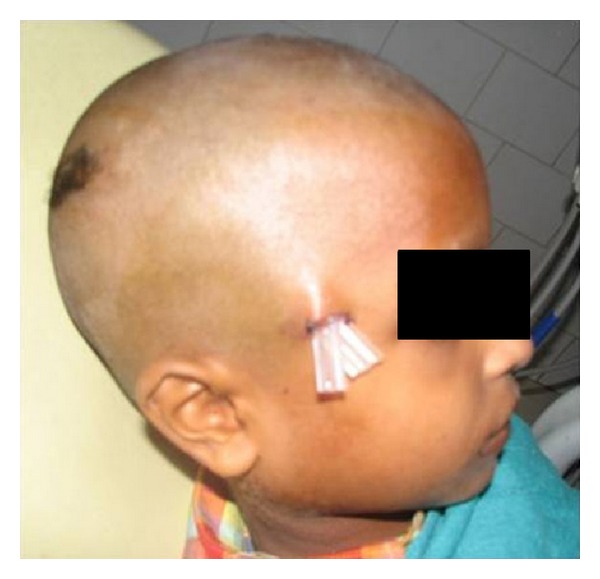
Postoperative picture after the second surgical intervention.

**Figure 4:**
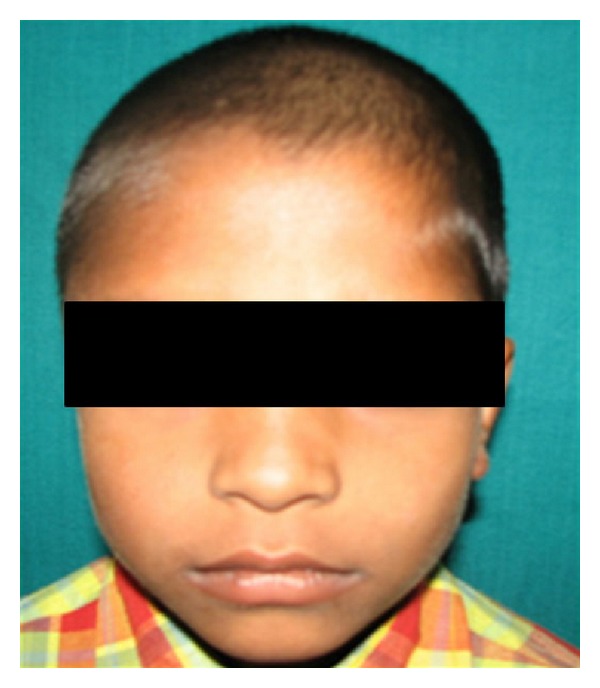
Two weeks postoperative picture of the patient.

 When the infection did not subside after 96 hours, another surgical exploration was planned under general anesthesia. A thorough exploration was carried out, the remaining pus, fat, hematoma, and necrosed fascia were removed, and the antibiotics were changed according to culture and sensitivity reports. The patient was advised mouth opening exercises after 24 hrs as his mouth opening had decreased considerably (Figure 3). As the condition of the patient improved, the patient was discharged with advice to continue mouth opening exercises under sensitive antibiotic coverage for two weeks. Subsequently the patient recovered completely and has been followed for last 14 months without any complication with a mouth opening of 38 mm. A final diagnosis of temporal space infection was established (Figure 4). 

## 3. Discussion

Trismus, severely restricted mouth opening, may occur as a result of intracapsular pathology of the temporomandibular joint or as a result of extracapsular pathology. Intracapsular causes of trismus are ankylosis, arthritis synovitis, meniscus pathology, and so forth. Extracapsular causes of trismus can be odontogenic (pulpal, periodontal), nonodontogenic (peritonsillar abscess, brain abscess, tetanus), trauma (mandibular fracture, ZMC fracture), tumor and oral care, radiotherapy and chemotherapy, drug-related (phenothiazine, halothane), congenital (hypertrophy of coronoid) mandibular nerve blocks (postinjection trismus due to infection and hematoma) [4]. 

Sawhney [2] has classified TMJ ankylosis in children based on OPG findings and identified four types in which type 1 is described as fibrous adhesions [5]. Our diagnosis was also of fibrous ankylosis which was based on the history of trauma to the right side of jaw, gradual reduction in mouth opening, restricted movements of the right condyle with no pain, and swelling, and the OPG of the patient revealed slight reduced joint space of right side.

 A variety of techniques for the treatment of TMJ ankylosis have been described including intraoral coronoidectomy, ramus osteotomy, high condylectomy, forceful opening of the jaw under general anesthesia, autogenous costochondral graft (CCG), and free vascularized whole-joint transplants [6]. Based on the diagnosis and age of the patient, it was decided to do a forceful mouth opening under general anesthesia, and the patient was further instructed to do aggressive physiotherapy following Kaban's seventh protocol.

The patient reported back with clinical signs and symptoms of right temporal space infection. We tried to rule out all probable causes of temporal space infections and came to the hypothesis that organized hematoma formation in the temporal muscle region due to recurrent trauma to temporalis muscle during aggressive physiotherapy may be the cause which got infected later.

 Organized hematoma develops in several stages. Initially, blood accumulates and chronic hematoma changes to organized hematoma through angiogenesis and neovascularization, as has been reported for subdural hematoma. Fibrosis also occurs. The causes of initial bleeding are various, such as facial trauma, postoperative bleeding, and vessel injury [7]. For our patient also recurrent hematoma due to aggressive physiotherapy causing overstretching of temporalis muscle leading to intramuscular bleeding, injury, and inflammation of the muscle may be the cause. 

 We further try to find the cause due to which hematoma got infected. After ruling out all the probable reasons which may have leaded to infected hematoma, we hypothesized that anchoretic infection may be the cause. Anachoresis is defined as preferential collection or deposit of particles at a site, as of bacteria or metals that have localized out of the bloodstream in areas of inflammation [8]. Though the patient was free of any clinical infection, subclinical infections which are mostly present in pediatric age group may be the reason for the anachoresis leading to infection of the hematoma. We treated the patient with incision, drainage, and removal of infected and necrosed material, according to the principles of surgical and antimicrobial infection management.

The patient was further advised to continue physiotherapy but this time under antibiotic coverage, and subsequently the patient's mouth opening increased and no further infection was reported.

 Thus the sequele was as follows:

 trauma→hematoma→trismus due to fibrous ankylosis→forceful mouth opening and active physiotherapy→hematoma formation in temporal mucle region→hematoma getting organized and vascularized→anachoretic infection reaching hematoma due to increased vascularity→trismus due to temporal space infection→surgical intervention→active physiotherapy under antibiotic coverage→increased mouth opening.

## 4. Conclusion

The important factor that one needs to bear in mind with cases of trismus is that physiotherapy that includes unassisted and finger-assisted stretching exercises, and the Ferguson mouth gag when initiated should be gradual and of mild to moderate intensity with antibiotic cover to avoid any future unexpected complications. We advocate that further studies be done on complications of forceful mouth opening and active or passive physiotherapy. We also advocate that a standard protocol be formulated for the patients requiring mouth opening physiotherapy with emphasis on how much mouth opening to be done at what time and stage, to prevent any damage or complication to the surrounding structures involved during the physiotherapy.
